# CT-based patient-specific instrumentation for total knee arthroplasty in over 700 cases: single-use instruments are as accurate as standard instruments

**DOI:** 10.1007/s00167-020-06150-x

**Published:** 2020-07-16

**Authors:** Stefan Gaukel, Raphael N. Vuille-dit-Bille, Michel Schläppi, Peter P. Koch

**Affiliations:** 1 Department of Orthopedics and Traumatology, Cantonal Hospital Winterthur, Brauerstrasse 15, 8409 Winterthur, Switzerland; 2 Department of Pediatric Surgery, University Children’s Hospital of Basel, Basel, Switzerland

**Keywords:** Total knee arthroplasty, Patient-specific instrumentation, Patient-specific instruments, Component positioning, Single-use instruments

## Abstract

**Purpose:**

Efforts in total knee arthroplasty are made to improve accuracy for a correct leg axis and reduce component malpositioning using patient-specific instruments. It was hypothesized that use of patient-specific instruments (vs. computer-navigated and conventional techniques) will reduce the number of outliers. Our second hypothesis was that single-use instrumentation will lead to the same accuracy compared to patient-specific instruments made of metal.

**Methods:**

708 primary total knee arthroplasties between 2014 and 2018 using computer tomography (CT)-based patient-specific cutting block technique and a preoperative planning protocol were retrospectively reviewed. Preoperative data [hip–knee–angle (HKA), lateral distal femoral angle (LDFA), medial proximal tibial angle (MPTA), tibial slope, femoral component flexion] was compared to postoperative performed standard radiological follow-up X-rays. Differences of > 3° between measurements were defined as outliers.

**Results:**

Overall 500 prostheses using standard instrumentation and 208 prostheses using single-use instruments were implanted. Preoperative HKA axes (− 1.2°; *p* < 0.001), femoral component flexion (Δ 0.8°, *p* < 0.001), LDFA (Δ − 1.5°, *p* < 0.001), MPTA (Δ − 0.5°, *p* < 0.001) and tibial posterior slopes (Δ 0.5°, *p* < 0.001), respectively, were different from postoperative axes. More outliers occurred using standard (vs. single-use) instruments (*p* < 0.001) regarding postoperative HKA (ranges of standard- vs. single-use: instruments: HKA 178.0°–180.5° vs. 178.0°–180.5°, femoral component flexion 0.0°–6.0° vs. 0.0°–4.5°, LDFA 90.0°–91.0° vs. 90.0°–90.0°, MPTA 90.0°–90.0° vs. 90.0°–90.0°, tibial posterior slope − 10° to 10° vs. − 1° to 10°). No differences were seen for other angles measured. Comparing both systems, total number of outliers was higher using standard (8%) vs. single-use instruments (4.3%).

**Conclusion:**

This study shows a high accuracy of CT-based patient-specific instrumentation concerning postoperative achieved knee angles and mechanical leg axes. Single-use instruments showed a similar accuracy.

**Level of evidence:**

III.

## Introduction

Malalignment of total knee arthroplasty (TKA) component positioning typically results in earlier component loosening and shortened survivorship of the prosthesis [1–9]. Deviations of more than 3° from neutral axis in the hip–knee–ankle axis (HKA) result in varus or valgus malalignment and are defined as ‘outliers’ when following an approach of classical mechanical alignment. Efforts were made to improve accuracy for a correct leg axis and reduced malalignment as well as component malpositioning by the invention of computer-assisted surgery (CAS) and patient-specific instruments (PSI). The main goal is to achieve a high accuracy in postoperative component positioning as compared to the preoperative planning. Multiple studies showed better results in terms of positioning and limb axis for CAS TKA compared to manual (i.e., conventional) instrumentation [2, 10–13]. Nevertheless, CAS TKA prolongates operating time and has shown problems arising from the guidance pins [14]. Other studies have shown promising results using PSI to achieve a lower rate of outliers [15–19]. To date, the evidence is rising from large-scale long-term studies comparing preoperative planning of PSI axis (i.e., hip–knee–ankle axis (HKA), lateral distal femoral angle (LDFA), medial proximal tibial angle (MPTA), the femoral component flexion and the tibial posterior slope) vs. postoperative resulted positioning [20]. More recently, single-use disposable instrumentation has been introduced to reduce costs by increasing efficiency in the operating room [21]. The combination of single-use instrumentation and PSI is, therefore, a promising approach, but so far information about its accuracy is lacking.

This study hypothesizes that use of PSI (vs. computer-navigated and conventional techniques) will reduce the number of outliers. The second hypothesis was that the combination of single-use instrumentation with PSI will lead to the same accuracy of component positioning compared to standard instrumentation with PSI.

## Materials and methods

### Materials

All consecutive knee replacement surgeries performed at one institution from January 2014 until December 2018 were retrospectively reviewed. All procedures were performed by two staff surgeons or under their direct guidance. The prospectively collected radiographic data according to the routine follow-up protocol were retrospectively analysed. The study was approved by the local ethics committee (Reference no. 2020-00198).

#### Inclusion criteria

All consecutive patients who underwent primary total knee arthroplasty using a CT-based patient-specific cutting block technique (GMK MyKnee©, Medacta International S.A., Castel San Pietro, Switzerland) with two types of prostheses using PSI (Medacta GMK MyKnee PSI stemless and GMK Efficiency PSI stemless, Medacta International S.A.) as well as a preoperative planning protocol were included.

#### Exclusion criteria

All patients with hinged prostheses (*n* = 30) as well as long stems (*n* = 109) and those with missing follow-up radiographic examinations in the first postoperative days (*n* = 8) were excluded. Furthermore, those with simultaneous high tibial osteotomy (*n* = 2) and concomitant knee diseases (i.e., hereditary multiple osteochondromas) (*n* = 4) were excluded.

### Workflow

The data of the preoperatively assessed standardized CT scans were sent to the company (Medacta International S.A.), where engineers planned the position and size of the TKA and returned the protocol back to the surgeon, who evaluated and corrected the plan over a three-dimensional (3D) online planning tool. In all cases a neutral mechanical (0°, i.e., HKA = 180°) or a constitutional axis (1°–2° varus alignment), a posterior slope of the tibia component between 0° and 5° and a flexion of the femoral component of 0°–4° were intended. LDFA and MPTA were planned as 90° to the HKA axis. Overall, the planning respected individual constitution, so that a large varus alignment was attempted to achieve a slight varus afterwards. After confirmation of the plan by the surgeon, patient-specific cutting blocks were manufactured using laser sinter technology. The planning protocol itself was stored on the Medacta server so that all angles, preoperatively measured by the engineers and planning software with the standardized CT scans and 2D/3D reconstructions, were available for later download and analyses.

**Fig. 1:**
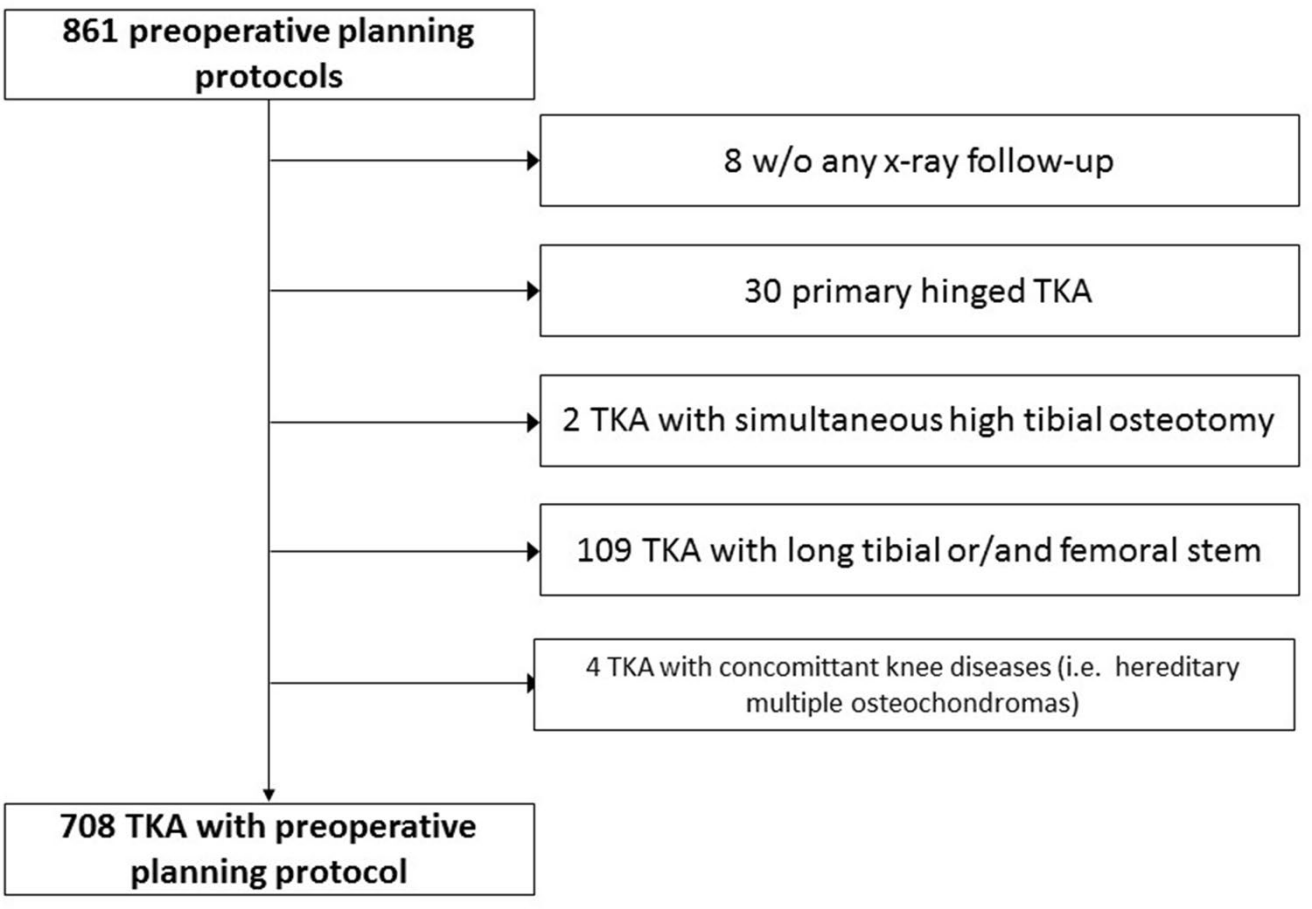
Flowchart for case selection. *TKA* total knee arthroplasty, *w/o* without

The following cases were excluded (see Fig. 1 for patient flow chart): 8 cases for missing long standing X-ray follow-ups, 30 cases for receiving a primary hinged prosthesis, 2 TKAs for simultaneous high tibial osteotomies, 109 cases for receiving additional long femoral and/or tibial stem, and 4 TKAs with concomitant diseases (i.e., hereditary multiple osteochondromas). The cases with additional stem were excluded as stem implantation has the potential of misguiding the planned component position independent to PSI.

### Radiological assessment

**Fig. 2:**
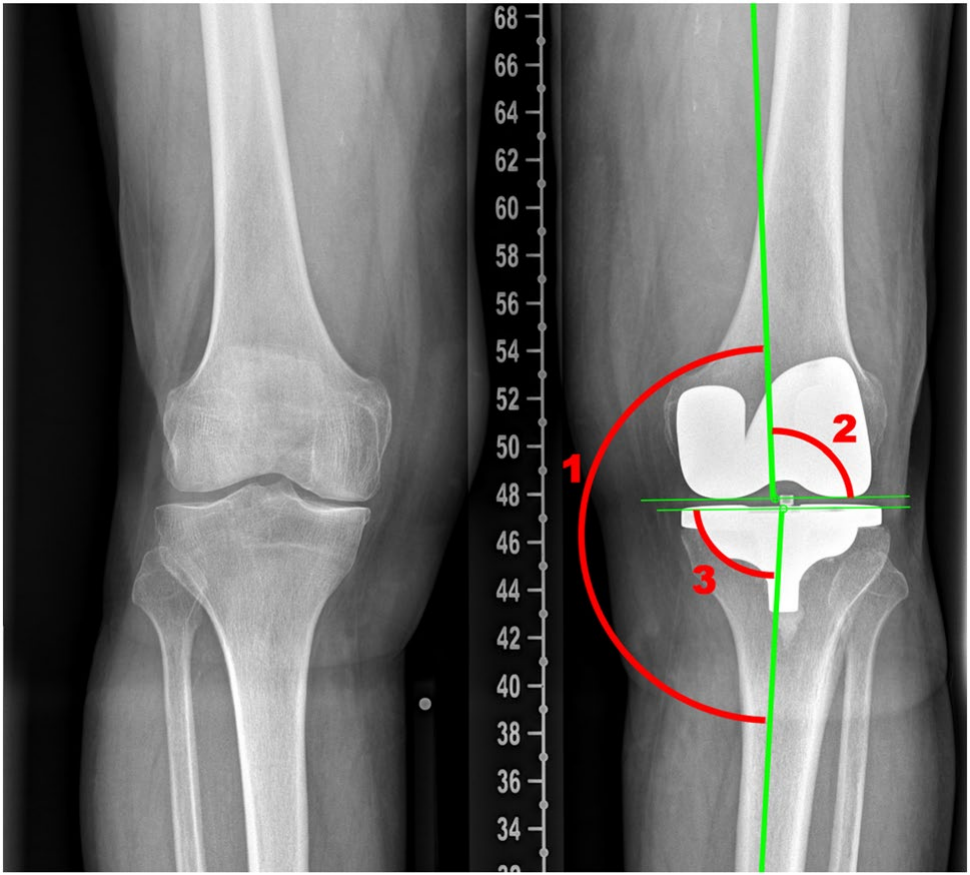
Measured angles on anterior–posterior view X-ray—HKA (1), LDFA (2) and MPFA (3)

**Fig. 3:**
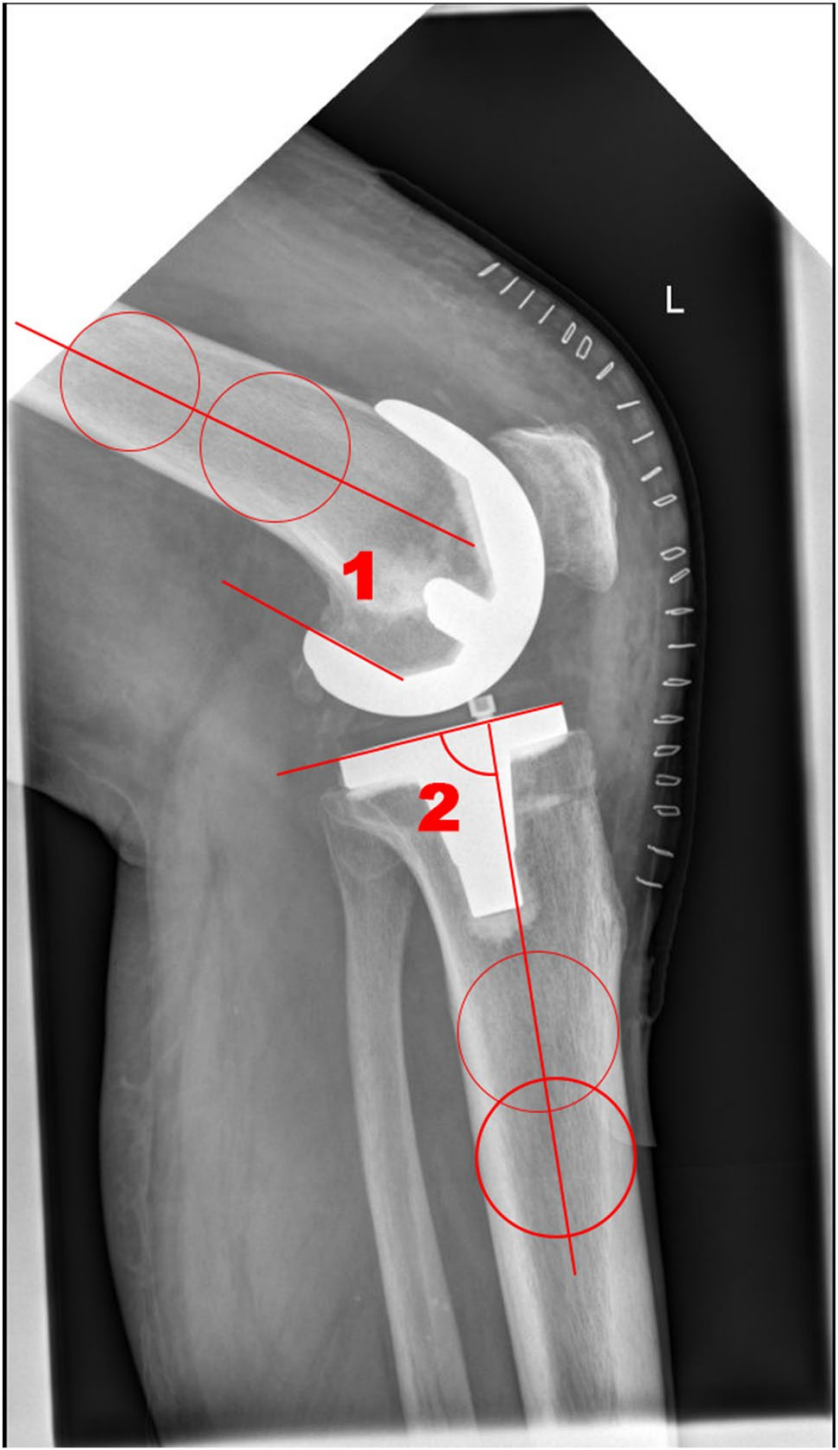
Measured angles on lateral view X-ray—femoral component flexion (1) and tibial slope (2)

For preoperative data all planning data consisting of HKA, LDFA and MPTA as well as tibial slope and femoral component flexion was exported and compared to postoperative values. The radiological assessment consisted of postoperative X-rays of the knee (lateral) in the first 2–4 postoperative days for tibial slope and femoral component flexion of the corresponding components. Long leg full weight-bearing X-rays 6–8 weeks after surgery as routine radiography during the first outpatient consultation were used for the HKA. The mechanical axis of the lower limb as well as the LDFA and the MPTA were measured in the postoperative long leg X-ray by the first author (SG), who was not involved in the surgery performed. Measuring was performed as shown in images 1 and 2. HKA was measured as the mechanical axis from the centre of the femoral head to the centre of the distal femur and the centre of the tibia plateau to the centre of the ankle joint in the frontal plane. LDFA was measured as the angle between the mechanical axis of the femur and the knee joint line of the femur in the frontal plane. MPTA was measured as the angle between the tibial knee joint line and the mechanical axis of the tibia in the frontal plane. The femoral component flexion was measured on the sagittal plane as the angle between the anatomical axis of the femur and a line drawn at the posterior condyle of the femoral component. The tibial slope was measured on the sagittal plane as the angle between the lateral tibial knee joint line and the tibial anatomical axis (See Figs. 2 and 3 for visualization).

All measurements were then compared to the preoperative planning done within the company’s online planning tool. Differences of > 3° in femoral component flexion, tibial slope, LDFA, MPTA and HKA were defined as outliers.

### Power analysis

To be able to compare the two cutting block models, 760 patients were calculated to obtain a statistical power of 0.85. The alpha error was set at 0.05. The effect size was set at 0.13 to show a statistical difference of 3%.

### Statistical analysis

All analyses were conducted using the software package R (Windows version 3.6.1; The R Project for Statistical Computing) with a significance level set at *p* < 0.05. Statistical comparisons were made using the Wilcoxon test for pre- and postoperative angles. The Chi squared test was used to compare the number of outliers between standard instrumentation PSI and single-use instrumentation. Effect of the operating surgeon on component positioning accuracy was tested using a Wilcoxon rank sum test.

## Results

### Demographics

During the study period a total of 861 knee replacement surgeries with an online preoperative planning protocol were performed. According to the exclusion criteria, 708 cases were hence included and analysed (see Fig. 1).

309 female (43.6%) and 399 male (56.4%) patients underwent surgery at a mean age of 69.6 years (ranging from 38.5–95.3 years). Case distribution among the two operating/supervising surgeons was: surgeon A: 293 cases (41.4%), surgeon B: 415 cases (58.6%). 350 right- (49.4%) and 358 left-sided (50.6%) TKAs were performed. The median time between surgery and first postoperative knee X-ray was 2.5 days (ranging from 0 to 58 days) and was 58 days (ranging from 19 to 681 days) between surgery and postoperative long leg X-ray. Further demographic data are provided in Table 1.

**Table 1:** Demographics of included cases

Female/male	309/399 (43.6%/56.4%)
Age (median and range) (years)	69.6 (38.5–95.3)
Right-sided/left-sided TKA	350/358 (49.4%/50.6%)
Surgeon A/surgeon B	293/415 (41.4%/58.6%)
Time between surgery and postoperative knee X-ray (median and range) (days)	2 (0–58)
Time between surgery and postoperative long leg X-ray (median and range) (days)	58 (19–681)

*TKA* total knee arthroplasty

### Radiological accuracy

The preoperative HKA axes differed from the postoperative ones (median delta − 1.2° [− 1.7°; − 0.8°], 176.4° (161.5°–190.5°) vs. 177.6° (169.0°–184.0°), *p* < 0.001). Likewise, the median delta of femoral component flexion (0.8° [0.5°; 1.0°], *p* < 0.001), the delta of the LDFA (− 1.5° [− 1.5°; − 1.49°], *p* < 0.001), of the MPTA (− 0.5° [− 0.5°; − 0.1°], *p* < 0.001), and of the tibial posterior slope (0.5° [0.5°; 0.75°], *p* < 0.001) were significantly different. For detailed information see Table 2.

**Table 2:** Results of preoperative planning and postoperative measurement

Knee angle	*N*	Preoperative planning median (range) (°)	Postoperative median (range) (°)	Δ median (°)	*p* value	95% confidence interval (°)	# Outliers (Δ median > 3°)
HKA axis	685	179.0 (178.0–180.5)	178.0 (169.0 to 184.0)	− 1.2	< 0.001	[− 1.7 to − 0.8]	54^a^ [7.9%]
Femoral component flexion	708	2.0 (0.0–6.0)	1.0 (−7.0 to 8.0)	0.8	< 0.001	[0.5 to 1.0]	96 [13.6%]
LDFA	685	90.0 (90.0–91.0)	91.0 (87.0 to 99.0)	− 1.5	< 0.001	[− 1.5 to − 1.49]	53 [7.7%]
MPTA	685	90.0 (90.0–90.0)	90.0 (86.0 to 96.0)	− 0.5	< 0.001	[− 0.5 to 0.1]	16 [2.3%]
Tibial posterior slope	708	3.0 (2.0–5.0)	3.0 (− 10 to 10)	0.5	< 0.001	[0.5 to 0.75]	86 [12.2%]

*HKA* hip–knee–ankle angle, *LDFA* lateral distal femoral angle, *MPTA* medial proximal tibial angle

^a^6 Patients (0.87%) preoperative > 180.0° and afterwards < 180.0° with delta > 3°. 2 patients (0.29%) preoperative < 180.0° and afterwards > 180.0° with delta > 3°

No effect of the operating surgeon on the component positioning accuracy was detected (*p* = 0.12).

Overall, there were 49 of 708 outliers (6.9%) in HKA (< 177.0° or > 183.0°), 96 outliers (13.6%) in femoral component flexion, 53 in LDFA (7.7%), 16 in MPTA (2.3%) and 86 in tibial slope (12.2%).

There were 6 patients (0.87%) with initial valgus HKA resulting in a postoperative varus HKA. Furthermore, there were 2 patients (0.29%) with a preoperative varus HKA resulting in a postoperative valgus HKA besides being planned to varus axis of 179.0° (both patient with postoperative 184.0°).

500 prostheses using the standard instrumentation PSI (70.6%) and 208 prostheses using the single-use instruments (29.4%) were implanted. There were more outliers using the standard instruments PSI than the single-use ones regarding postoperative HKA (*p* < 0.004). There were no differences regarding tibial posterior slope, femoral component flexion, LDFA and MPTA.

Even though the same surgical technique was used, significantly less cases were outside of the postoperative ± 3° range when using single-use PSI instrumentation as compared to standard instruments. For detailed numbers of outliers concerning the different measurements see Table 3.

**Table 3:** Percentage of outliers between preoperative planning and postoperative outcome

Knee angle	Standard instrument PSI group (*n* = 500) (%)	Single-use PSI group (*n* = 208) (%)	*p* value
HKA axis	49/483 (11.3)^a^	6/206 (3.0%)^b^	0.004
Femoral component flexion	70/500 (14.0)	26/208 (12.5)	0.730
LDFA	32/484 (6.6)	21/201 (10.4)	0.157
MPTA	10/484 (2.1)	6/200 (3.0)	0.667
Tibial posterior slope	63/499 (12.6)	23/208 (11.1)	0.697

*HKA* hip–knee–ankle, *LDFA* lateral distal femoral angle, *MPTA* medial proximal tibial angle

^a^49 Outliers with 2 preoperative > 180°/postoperative < 177° and 9 preoperative < 180°/postoperative > 183°

^b^6 Outliers with 1 preoperative > 180°/postoperative < 177° and 0 preoperative < 180°/postoperative > 183°

Comparing both systems used showed a lower total rate of outliers concerning all measurements combined in the single-use instruments (7/208 (4.3%)) than in the standard instrumentation PSI (40/500 (8.0%)) group (*p* = 0.049).

## Discussion

The most important findings of the present study were that CT-based patient-specific cutting blocks in total knee arthroplasty enable accurate postoperative angles and mechanical axes of the leg with a small number of outliers > 3° and single-use instruments showed no inferiority to standard instrumentation. This is the largest cohort of patients, where preoperative planning vs. postoperative radiological outcomes and standard vs. single-use instrumentation PSI were compared.

Compared to previously published data regarding the accuracy of PSI this study was able to confirm the good postoperative results using the MEDACTA PSI technology with a smaller rate of outliers including twice as much patients [18]. The authors gained a rate of outliers for HKA of 11.7% which was lower in the present analysis (7.9%). Similar results for the other angles measured were achieved. The reduction of outliers in the present data can possibly be explained by the learning curve of the surgeon (PK) performing the arthroplasties back then as compared to the present study.

Hereby presented results in postoperative angles and HKA showed very small (e.g., as maximum up to 1.5° for LDFA), but statistically significant differences from the preoperative planning on the manufacturer’s online tool. It is questionable, whether these statistical differences are of any clinical relevance or not. This difference is lying within the measurement accuracy especially for the HKA, tibial slope and the femoral component flexion. Radtke et al. showed in their study about the effect of limb rotation on radiographic alignment in total knee arthroplasties that limb rotation had a significant effect on measured anatomic alignment and mechanical angles. LDFA, for example, differed up to 4° with limb rotation within a range of 20° with increasing LDFA and MPTA during external rotation and vice versa [22]. Therefore, small differences between preoperative planning with CT scan and postoperative measurements on X-rays must be viewed with this possible measuring error kept in mind. The tibial slope and femoral component flexion angles were, furthermore, sometimes difficult to measure accurately because of a lack of a long displayed bone shaft on the X-ray pictures. In our opinion a slight constitutional planning, i.e., 1°–2° varus, can prevent an overcorrection of the HKA to valgus axis, as shown on the low rate of overcorrected HKAs in the present study.

Compared to published data dealing with PSI accuracy, the present results are comparable to published data of CAS TKA and show more favourable results as compared to conventional techniques [16, 23–25]. Using conventional techniques, studies report only 28–85% of cases achieving a mechanical leg alignment within the 3° varus/valgus range [11]. Chan et al. postulated that even in patients with BMI > 30 kg/m^2^ PSI showed a higher accuracy than the conventional technique [16]. A meta-analysis performed by Hetaimish et al. [26] compared conventional TKA to CAS TKA. They showed a higher rate of outliers compared to the hereby presented PSI group for both, conventional TKA (30.1%) as well as CAS TKA (12.8%). Furthermore, their rates of outliers for LDFA (16.4% vs. 7.2%) and MPTA (12.4% vs. 5.8%) were higher than in the present cohort of patients. Another meta-analysis performed by Cheng et al. [23] including 15 RCTs and 26 quasi RCTs comparing conventional technique vs. TKA confirmed these rates of outliers for both techniques.

A slightly lower number of outliers when using CAS TKA compared to hereby presented results for PSI could be shown by Tingart et al. [27] who analysed 100 prospective cases of computer-assisted vs. conventional TKAs with an outlier rate of 5 vs. 26% and a mean deviation from neutral axis of 1.6° vs. 2.3°. Other authors agree with their critical objection towards the PSI systems with results of only slight improvements in accuracy of component placing compared to other techniques [14].

Anderl et al. [15] included in their prospective study 300 knees to compare conventional vs. patient-specific instrumentation regarding postoperative radiological limb alignment and component positioning. Their CT-based PSI group showed a better component alignment and especially for the HKA a better postoperative result than those knees operated with the conventional technique. Thienpont et al. [13] performed a systematic review of published data for conventional, computer-navigated and patient-specific instrumented TKA which showed a superiority of CAS over conventional techniques but a lack of studies with high quality and sufficient power for the use of PSI. Only one study was able to show a lower rate of outliers when using PSI instrumentation [19]. Therefore, this work intends to publish our data on CT-based PSI in TKA and are hereby presenting improved radiological outcomes in a large cohort of patients.

There are some published studies comparing single-use instrumentation vs. standard metal instrumentation PSI and conventional techniques, respectively. Abane et al. [28] showed similar radiological outcomes for all three techniques. For HKA they showed an outlier rate of 24% compared to 22% in the standard metal instrumentation PSI group and 20% when using conventional technique. The hereby presented rate of outliers concerning HKA was much lower with 11% (standard metal instrumentation) vs. 3% (single-use instrumentation), respectively. Furthermore, this study was able to show a lower rate of outliers concerning the other measured angles. Gianotti et al. included only 40 patients in their study comparing conventional instrumentation vs. single-use PSI. But they also confirmed the equality between both techniques when analysing postoperatively achieved HKA axes [29].

Less than 7% of included patients were outliers regarding HKA axis with > 3° deviation, which may be a risk factor of early component failure as shown by Berent et al. and other authors regarding tibial component failure mechanism after TKA [1, 5, 8, 9]. Jiang et al. analysed 18 studies (10 using CT-based and 8 MRI-based systems) including 2417 patients in total and did not show superiority in accuracy of component placing when using PSI [17]. The overall outlier rate in HKA for PSI (17.4%) compared to the conventional technique (19.3%) was much higher than in the hereby included population. On top of that, all other measured angles in this population had lower outlier rates for PSI than shown in this review (femoral component flexion 26%, tibial slope 23.7%, LDFA 5.8%, MPTA 5.5%).

Different studies in the past were able to show that an accurate component positioning results in a long durability of the prosthesis [3, 4, 9]. With the small degree of deviation from the original planning protocol almost all of included cases were lying within the published range for a long survivorship of the components. On the other hand, Parratte et al. stated that the narrow definition of a perfect component alignment within 0° ± 3° does not affect the component’s survivorship over a 15 year observation time and proposed a shift in the dichotomous use of these variables for predicting the durability of modern TKA [30]. The same opinion was shared by Bonner et al. [2] who analysed 501 TKA grouped into an aligned and mal-aligned cohort with HKA > 3° valgus/varus and found only a weak relationship between alignment and survivorship of components. With new kinematic alignment strategies, this shift away from the 3° range for HKA is under development nowadays. Nevertheless, accuracy remains a main issue to achieve the planned alignment.

One has to keep in mind that for the accuracy of PSI systems the new technology itself depends not only on the technology of patient-matched cutting blocks and preoperative CT or MRI scans but also on the technique and routine used to implant the components. Despite some authors believing that PSI doesn’t require any excessive learning curve [16], in our opinion and in other authors’ conclusion surgeons not only have to get used to a new implant technology but also must get familiar with a different implantation technique which may bias results [31]. Jiang et al. [17] conclude in their systematic review comparing PSI vs. conventional technique that the learning curve as well as the use of new instruments and getting used to the new technique may have a stronger effect than initially expected. Hereby presented results did not depend on the surgeon performing the operation. Therefore, no correlation with an individual learning curve for a better postoperative radiological outcome could be shown. This result can be explained by the high experience and an already advanced learning curve of the surgeons which were familiar with the technique using standard metal instruments and profited from this knowledge of the technique when switching to single-use instruments some years later.

Regarding the follow-up X-ray interval, there were two outliers: first, there was a follow-up at day 0 (i.e., X-ray at the day of surgery) due to persistent pain and reduction in general condition, leading finally to the diagnosis of a pulmonary embolism and referral to the colleagues of internal medicine. Postoperative X-ray of another patient was performed by the rehabilitation clinic he was transferred to and send to our clinic but could not be measured in our X-ray image viewer software. We, therefore, analysed the first postoperative X-ray in the outpatient setting performed after 58 days. Secondly, there was a long leg X-ray in the outpatient setting performed after 681 days as a single outlier from the routine protocol. No long leg X-ray on the 6 week outpatient consultation existed for further analysis, so that the first long leg X-ray after arthroplasty on the contralateral side was taken for the measurements.

### Limitations of the present study

Despite being limited by the retrospective study design, a large number of patients was included in the present study. Because of a change towards single-use instrumentation PSI since June 2017, hereby obtained results might be biased by other factors that might have changed over time. Another limitation of the present study is the fact that measurements of postoperative angles were only performed by one person. Furthermore, potential confounding factors for HKA measurements are the rotation of the limb as well as extension or flexion limits of the knee in long leg X-rays. Lonner et al. [32] previously described the influence of malrotation and flexion/extension limits of the knee on tibial and femoral measurements making objective evaluation sometimes difficult. No long leg X-ray was excluded from this analysis to reduce a possible data selection bias.

In addition, all measurements were only done on conventional X-ray films, and especially tibial slope and femoral component flexion were sometimes difficult to measure on plain X-ray films with only short displayed bone shafts for the determination of the bone axis. For exact measurements, CT scans as performed preoperatively would be needed [33]. But due to the irradiation exposure of the patients their performance would not be considered ethical. Recent studies provided good evidence that long leg full weight-bearing X-rays are reliable for assessing alignment of the HKA [34] with lower radiation exposure and high inter-observer and inter-modality correlation comparing X-ray to CT. Furthermore, almost all published studies measure the precision of TKA placement on conventional X-ray which makes the values comparable.

Another limitations of this study is the fact, that the measurements of the postoperative X-ray were only performed by one surgeon (SG) and no second observer measurement was available. Therefore, the measurements accuracy only depends on one single measurement. We tried to improve measurement accuracy by performing a second measurement of, e.g., femoral component flexion and tibial slope on the X-rays from the first outpatient visit and the comparison to the measurements of the postoperative values by chance. Furthermore, we used the automatic measurement feature of the prosthesis planning tool in our X-ray viewer program to control the measurements by chance and did not see any significantly differences.

Whereas the retrospective study design reflects a disadvantage and a possible source of bias, a strong advantage of the present study is the high number of included patients and the small number of surgeons performing the operation.

No clinical data of the included patients undergoing total knee arthroplasty was analysed. Hence, the clinical impact of the measured outliers was not assessed.

Nevertheless regarding clinical relevance of this study, it was shown that the use of patient-specific instrumentation in total knee arthroplasty leads to very accurate component positioning even when using single-use instruments instead of standard instruments made of metal.

## Conclusion

This study shows a high accuracy of CT-based patient-specific instrumentation concerning postoperative achieved knee angles and mechanical leg axes. Single-use instruments showed a similar accuracy.

## Abbreviations

CAS: Computer-assisted surgery
CT: Computer tomography
HKA: Hip–knee–ankle axis
LDFA: Lateral distal femoral angle
MPTA: Medial proximal tibial angle
MRI: Magnetic resonance imaging
PSI: Patient-specific instruments
TKA: Total knee arthroplasty
